# Combined Pre- and Postharvest Melatonin Treatments Improve the Functional Quality of the Sweet Cherry cv. ‘Sunburst’

**DOI:** 10.3390/foods14193337

**Published:** 2025-09-26

**Authors:** Fernando Garrido-Auñón, María Emma García-Pastor, María Serrano, Daniel Valero, Vicente Agulló

**Affiliations:** 1 Department of AgroFood Technology, EPSO-CIAGRO, University Miguel Hernández, Ctra. Beniel km. 3.2, 03312 Orihuela, Alicante, Spain; fgarrido@umh.es (F.G.-A.); daniel.valero@umh.es (D.V.); 2 Department of Applied Biology, EPSO-CIAGRO, University Miguel Hernández, Ctra. Beniel km. 3.2, 03312 Orihuela, Alicante, Spain; m.garciap@umh.es (M.E.G.-P.); m.serrano@umh.es (M.S.)

**Keywords:** anthocyanins, bioactive compounds, commercial quality, cold storage, hydroxycinnamic acids

## Abstract

Chronic metabolic disorders have increased recently due to changes in dietary habits and lifestyle. Red-coloured fruits, such as sweet cherries, are rich in anthocyanins and other (poly)phenolic compounds with health-promoting properties, which has garnered growing scientific interest. Melatonin elicitation has emerged as a promising strategy to improve the functional quality of these fruits. This research investigates, for the first time, the combined effect of pre- and postharvest melatonin treatments, followed by a cold storage (2 °C) of 21 days, on the endogenous melatonin and phenolic compound levels of 90 sweet cherries (*n* = 3) from the ‘Sunburst’ cultivar and harvested from 9 trees per treatment. Single preharvest or postharvest melatonin treatments increased the endogenous melatonin content via direct absorption and activation of key biosynthetic genes, while they reduced anthocyanin, hydroxycinnamic acid, and flavonol levels, likely due to a ripening-delaying effect at harvest. Nevertheless, the combined treatment increased endogenous melatonin levels 5-fold compared to harvest and increased all measured polyphenolic compound levels, including a 29% rise in total anthocyanins reverting the delay in the ripening process. These effects suggest upregulation of genes in the phenylpropanoid pathway and could improve fruit’s functional quality. The response to melatonin is cultivar- and dose-dependent. Future research should investigate genetic and transcriptomic validation to confirm these potential effects and assess whether increased bioactive compound content would translate into measurable human health benefits.

## 1. Introduction

In recent decades, the global burden of chronic metabolic disorders has escalated considerably, in parallel with profound changes in lifestyle and dietary habits. Conditions such as obesity, insulin resistance, and cardiovascular dysfunction have become increasingly prevalent across diverse populations, significantly contributing to morbidity and healthcare expenditures [1]. These diseases are frequently driven by multifactorial interactions between genetic predisposition, environmental pressures, and behaviour-related factors such as sedentary routines and psychological stress [2]. In this sense, integrated preventive strategies are required to combat these diseases [2]. It is increasingly evident that nutritional interventions, particularly those incorporating functional foods rich in antioxidant compounds, are recognized as essential components in addressing this public health challenge [3].

Fruit and vegetable consumption plays a pivotal role in such dietary interventions, given their high content of essential nutrients and phytochemicals, especially polyphenols, which have been shown to exert antioxidant, anti-inflammatory, and cardioprotective effects [4,5]. A number of epidemiological and mechanistic studies have indicated the capacity of these compounds to modulate redox balance, reduce low-grade inflammation, and attenuate oxidative damage [6]. This, in turn, has been demonstrated to contribute to the prevention of metabolic and degenerative pathologies [6]. In this context, red-coloured fruits such as berries and cherries, which are particularly rich in anthocyanins and other phenolics, have attracted increasing attention due to their high nutritional and functional value [7].

Sweet cherry (*Prunus avium* L.) is a fruit crop of economic and nutritional importance in Europe. In 2023, Spain was the second-largest producer in the region and the sixth largest worldwide [8]. Furthermore, national sweet cherry crop yield has increased by approximately 32% during 2024, accompanied by notable growth in both export volume and domestic demand [8]. This expansion can be attributed to an increasing consumer preference for fruits that offer added health benefits [9,10]. Among the available cultivars, Sunburst is highly regarded for its substantial size, dark red pigmentation, and elevated anthocyanin concentration. These characteristics not only contribute to its visual and organoleptic appeal but also enhance its phytochemical profile [11,12].

The health-promoting attributes of sweet cherries are largely attributed to their diverse array of phenolic compounds, which include anthocyanins (mainly cyanidin 3-*O*-rutinoside and cyanidin 3-*O*-glucoside), hydroxycinnamic acids, and flavonols [13]. These molecules have been demonstrated to contribute to the fruit’s high antioxidant activity [10,14,15]. In addition, there is a growing body of evidence associating them with potential benefits for the prevention of cancer, neuronal and cardiovascular diseases, diabetes and other inflammatory diseases [10]. However, the synthesis and accumulation of such compounds are strongly influenced by cultivar and rootstock combination, agronomic practices, and postharvest handling, particularly storage conditions and duration [16,17].

In this framework, a range of approaches have been investigated with a view to enhancing fruit quality and their associated health-promoting properties, with a primary focus on increasing the accumulation of phenolic compounds. Among these, chilling stress has been shown to trigger phenolic biosynthesis through the induction of phenylalanine ammonia-lyase (PAL) activity [18]. Consequently, fluctuations in temperature during the transportation of the cold chain have been observed to accelerate anthocyanin synthesis, phenolic metabolism, and degrade cell wall components of sweet cherry cultivar ‘Hongdeng’. These changes result in accelerated colour change and substantial fruit softening. The upregulation of specific genes and increased enzyme activity are associated with both colour development and structural deterioration [19]. On the other hand, melatonin (N-acetyl-5-methoxytryptamine) has emerged as a promising tool in horticulture, due to its potential to enhance the accumulation of phenolic compounds, such as flavonoids, which contribute to antioxidant capacity and overall fruit quality [20,21,22]. The exogenous application of melatonin has been the subject of extensive research on sweet cherry cultivars, at both the preharvest and postharvest stages. This research has demonstrated the ability of melatonin to enhance the biosynthesis of phenolic compounds, the activity of antioxidant enzymes, maintain reactive oxygen species (ROS) homeostasis, and delay senescence processes [23,24,25,26]. Nevertheless, the physiological response to melatonin treatments has been reported to be highly dependent on factors such as fruit species, cultivar, and treatment duration, resulting in variable outcomes [27]. The findings provide substantial evidence in support of the efficacy of a combination of cold storage and melatonin application as a strategy to enhance sweet cherry quality. This approach has been demonstrated to result in significant improvements in phenolic compound profiles and other functional properties.

To date, no study has comprehensively assessed whether the combined application of melatonin at both preharvest and postharvest stages, together with chilling cold stress, can simultaneously enhance the accumulation of phenolic compounds and stimulate endogenous melatonin biosynthesis in sweet cherry fruit. The objective of this study was to evaluate, for the first time, the impact of an integrated strategy on the levels of key phenolic subclasses (anthocyanins, hydroxycinnamic acids, and flavonols) and endogenous melatonin content in ‘Sunburst’ sweet cherries.

## 2. Materials and Methods

### 2.1. Plant Material and Experimental Design

The experiment was conducted using 7-year-old sweet cherry cv. ‘Sunburst’ trees (*Prunus avium* L.) grafted onto SL-64 rootstock in a commercial plot from the company “Mas de Roc Cooperativa Valenciana”, which is located in Alcoy (Alicante, Spain). During the experiment, 9 trees per treatment were selected at random for the preharvest treatments with melatonin at 0.5 mM, as well as for the control. A randomized block experimental design was set with three blocks of three trees (*n* = 3) for each treatment with a spatial block of 4 × 5 m. No relevant row and position effect were observed between trees. The selected melatonin concentration was based on previous experiments reported in the literature, which demonstrated that this dose elicited the greatest increase in phenolic content in sweet cherries [23,24,28,29].

Preharvest treatments consisted of foliar spraying of 1.5 L of the corresponding solution per tree using a mechanical mist sprayer to the tree canopy at two key stages: at pit hardening and at the beginning of anthocyanin biosynthesis (on 28 May and 6 June, respectively). After this, a period of two weeks elapsed before the harvesting of the fruits (19 June). Melatonin aqueous solutions were freshly prepared with analytical-grade melatonin (≥98% purity, Sigma-Aldrich, Madrid, Spain, CAS No. 73-31-4) dissolved in distilled water with 0.5% Tween-20 as a surfactant, yielding neutral pH solutions. The treatments were applied early in the morning and stored in an opaque container to minimize the potential photodegradation of melatonin by sunlight. Control trees received distilled water with the same surfactant.

At harvest, 90 homogeneous sweet cherries (10 fruits per tree) of uniform colour and size, free from visible defects or physical damage, were collected for each treatment. Fruits were then divided into three homogeneous groups of 30 fruits, corresponding to the following conditions: (1) Pretreatment at harvest (Pre-AH): 30 fruits were processed immediately at harvest for physicochemical and phytochemical analysis; (2) Cold storage (CS): 30 fruits were stored for 21 days at 2 °C and 85–90% relative humidity; (3) Postharvest treatment plus Cold Storage (Pre + Post + CS): 30 fruits were dipped in a 0.5 mM melatonin solution after harvest for 15 min, including Control fruits (as stablished in previous experiments [21,22,30]), and after air-drying at room temperature, these fruits were also stored under the same cold conditions as the CS group. Each group of 30 fruits was further subdivided into three subgroups of 10 fruits each, providing a biological replicate number of *n* = 3 for all subsequent analyses. It should be noted that the control fruits in the Pre + Post + CS group were subjected exclusively to the postharvest melatonin treatment. Melatonin application in postharvest treatments does not follow a single, fixed solution-to-fruit ratio, as optimal application is highly dependent on fruit type, maturity, and the desired quality outcome. Studies demonstrate that a range of melatonin concentrations is required to effectively delay ripening, enhance antioxidant activity, and improve fruit firmness [24,29]. For sweet cherries, postharvest treatments are typically conducted by immersing the fruit in an aqueous melatonin solution at ambient temperatures (20−25 °C). Gentle agitation during immersion ensures uniform coverage. Following treatment, the fruit was subjected to a 2.5 h air-drying period in the dark, after which it is stored at specific refrigerated conditions (e.g., 2 °C), to maintain quality and extend shelf-life. This methodology therefore focuses on concentration and environmental conditions rather than a volumetric ratio to achieve the desired physiological responses. The cold storage conditions employed are consistent with those commonly used in commercial practice to minimize postharvest quality losses in sweet cherries [29]. No visible decay incidence was detected during storage. Tissue preparation and analytical determinations of the CS and Pre + Post + CS groups were carried out immediately after the 21-day storage period.

### 2.2. Sample Processing

The cherries were then meticulously halved and the pits being extracted. The samples were subjected to a cryogenic process involving freezing in liquid nitrogen, followed by a freeze-drying procedure conducted under reduced pressure (2.2 MPa) for a duration of one day. This process was undertaken using an Alpha 2–4 freeze dryer (Christ Alpha 2–4; Braum Biotech, Melsungen, Germany). During this process, the drying chamber was maintained at a temperature of −25 °C, and the heating plate was set to 15 °C, in order to preserve their chemical composition in accordance with Badiche-El Hilali et al. [31]. After the freeze-drying process, the samples were ground into a fine powder, vacuum-packed, and stored at −20 °C until further analysis. The results were expressed on a fresh-weight basis (FW).

### 2.3. Extraction and Quantification of Melatonin Content

The sample processing method used to determine the melatonin content was the same as the one recently described by Agulló et al. [21,22]. Briefly, 100 mg of sweet cherry was mixed with 500 μL of dimethyl sulfoxide, and vortexed for 5 min. Subsequently, 1500 μL of a methanol/water (1:1, *v*/*v*) solution was added to the preceding mixture and subjected to mild ultrasonic treatment for a period of 10 min at room temperature. Finally, the samples were centrifuged at 10,500 rpm for 5 min (Sigma 1–13, B. Braun Biotech International, Osterode, Germany) and supernatants were filtered through a polyvinylidene fluoride (PVDF) filter with a diameter of 0.22 mm (Millex HV13, Millipore, Bedford, MA, USA). Prior to analysis, samples were stored at −20 °C. Melatonin determination and quantification was analyzed using a UHPLC-QqQ-MS/MS (UPCL-1290 Series and a 6460 QqQ-MS/MS; Agilent Technologies, Waldbronn, Germany) with an Acquity BEH C18 column (2.1 × 150 mm; 1.7 μm; Waters, Milford, MA, USA), following the same method as previously described [21]. Melatonin content was expressed as a concentration of ng per 100 g of FW of sample (ng/100 g).

### 2.4. Qualitative and Quantitative Analysis of Individual Phenolic Compounds

The characterization of phenolic compounds was performed using HPLC–MS/MS according to a previously established protocol [32]. Briefly, sweet cherry samples (prepared as described above) were analyzed in triplicate by chromatographic separation. A binary mobile phase composed of 1% formic acid in water (solvent A) and acetonitrile (solvent B) was employed. The gradient started at 15% B, increased to 30% over 20 min, to 40% at 30 min, 60% at 35 min, and 90% at 40 min. This composition was maintained for 4 min before reverting to the initial conditions, followed by a 10 min equilibration period. The chromatographic flow rate was set at 0.9 mL/min. Mass spectrometric detection covered an m/z range of 100–1200, with operating parameters as follows: capillary temperature 350 °C, voltage 4 kV, nebulizer pressure 65.0 psi, and nitrogen flow 11 L/min. For MS acquisition, the flow rate was adjusted to 0.8 mL/min, and solvent gradients were applied linearly. Collision-induced dissociation (CID) was carried out in an ion trap with helium as the collision gas, applying voltage ramps from 0.3 to 2 V. Anthocyanins were analyzed in positive electrospray ionization mode, while non-colored phenolic compounds were monitored in negative mode. Multistage MSⁿ analyses were automatically triggered for the most abundant product ion obtained in the preceding MS^n−1^ spectrum. Instrument control and data processing were managed with ChemStation for LC 3D Systems Rev. B.01.03-SR2 (Agilent Technologies Spain S.L., Madrid, Spain).

Quantification of phenolic compounds was performed by HPLC–DAD following previously reported methodologies [21,33,34]. Analyses were conducted on a Luna C18(2) column (250 mm × 4.6 mm, 5 µm, 100 Å) with a Security Guard Cartridge (4 mm × 3.0 mm) (Phenomenex, Torrance, CA, USA). An Agilent Technologies 1220 Infinity HPLC system, equipped with an autosampler (G1313) and a diode array detector (model 1260, Agilent Technologies, Santa Clara, CA, USA), was used. The mobile phase consisted of solvent A (Milli-Q water with 5% formic acid, *v*/*v*) and solvent B (methanol). The gradient program started at 15% B, increased to 30% at 20 min, 40% at 30 min, 60% at 35 min, and reached 90% at 40 min. This composition was held until 44 min, then returned to 15% B at 45 min and maintained until 50 min to re-equilibrate the column. Injection volume was 10 µL, with a constant flow rate of 0.9 mL/min. Data acquisition and processing were performed using ChemStation for LC 3D Systems software v. 4.1 (Agilent Technologies). Anthocyanins, hydroxycinnamic acids, and flavonols were quantified as cyanidin-3-*O*-glucoside (wavelength of 520 nm), chlorogenic acid (wavelength of 320 nm), and rutin (wavelength of 360 nm), respectively. These concentrations were expressed as milligrams per 100 g of FW of the sample (mg/100 g).

### 2.5. Statistical Analysis

The results were presented as means ± SD (*n* = 3). The statistical differences between treatments at the same conditions (control vs. melatonin treated) were identified through the application of *t*-test. Regarding the statistical differences between conditions for the same treatment (Pre-AH vs. CS vs. Pre + Post + CS), they were identified through the application of one-way analyses of variance (ANOVA) and the multiple range test of Tukey. To control Type I error, analyses were performed separately for each compound (melatonin, anthocyanins, hydroxycinnamic acids, and flavonols), and the family-wise error rate was therefore considered within each compound. The level of statistical significance was set at *p* < 0.05. All statistical analyses were performed using SPSS 17.0 software (LEAD Technologies, Inc., Chicago, IL, USA). The PCA model was also constructed with normalized data using the version 17.0 of SPSS software package.

## 3. Results

### 3.1. Impact of Melatonin Treatments on Melatonin Accumulation in Sweet Cherry Fruit

**Figure 1:**
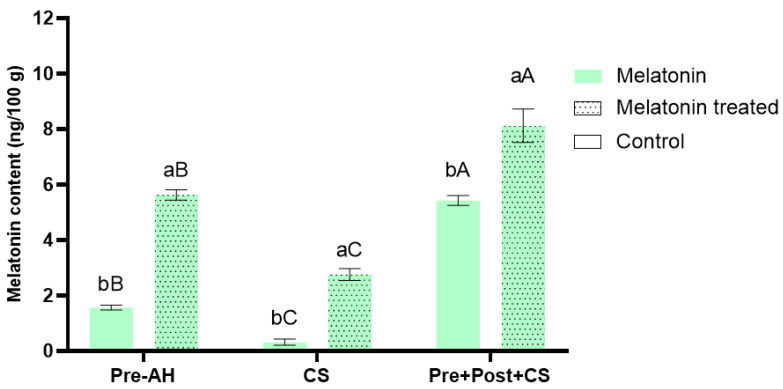
Endogenous melatonin content (ng/100 g) in sweet cherries for the different treatments (control and melatonin) and conditions (preharvest; Pre-AH, cold storage; CS, and preharvest plus postharvest treatment followed by cold storage; Pre + Post + CS). Data is presented as mean ± Standard Deviation (SD). Control group is represented by the bars not filled with dots and melatonin treated group is represented with bars are filled in. Distinct lowercase letters denote statistically significant differences between treatments determined using *t*-test (*n* = 3) for each sampling date. Distinct uppercase letters denote statistically significant differences between conditions determined using one-way analysis of variance (ANOVA) followed by Tukey’s multiple range test (*n* = 3) for each sampling date. A significance level of *p* < 0.05 was set for both statistical analyses.

The endogenous melatonin content in sweet cherry fruits under all treatment conditions was determined by HPLC-QqQ-MS/MS (Figure 1).

The application of exogenous melatonin to sweet cherries resulted in a significant increase in endogenous melatonin content compared to control fruits under all evaluated conditions. At harvest, melatonin-treated fruits increased their content approximately 3.6 times compared to controls (5.63 ± 0.19 vs. 1.57 ± 0.09 ng/100 g, respectively). However, cold storage led to a significant reduction in endogenous melatonin levels, by 80% in control fruits and 51% in melatonin-treated fruits. When postharvest melatonin treatments were combined with cold storage, control fruits showed the greatest relative increase, approximately 3.5 times higher than control fruits at harvest (5.43 ± 0.17 ng/100 g). Furthermore, the combination of pre- and postharvest treatments followed by cold storage led to the highest absolute values among all conditions (8.12 ± 0.61 ng/100 g), exhibiting a 5-fold increase compared to control at harvest.

### 3.2. Impact of Melatonin Treatments on Anthocyanins Accumulation in Sweet Cherry Fruit

**Figure 2:**
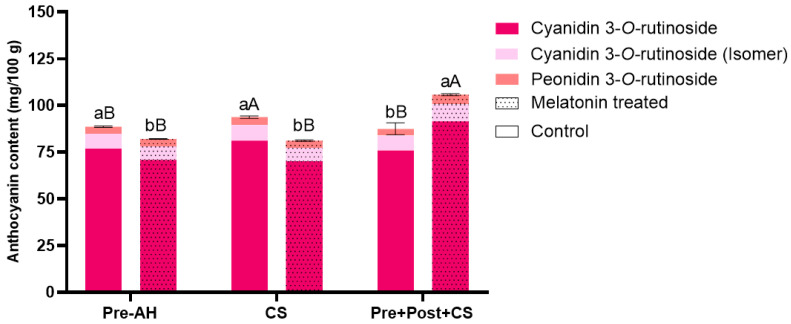
Anthocyanin content (mg/100 g), specifically cyanidin 3-*O*-rutinoside, cyanidin 3-*O*-rutinoside (Isomer) and peonidin 3-*O*-rutinoside, in sweet cherries for the different treatments (control and melatonin) and conditions (preharvest; Pre-AH, cold storage; CS, and preharvest plus postharvest treatment followed by cold storage; Pre + Post + CS). Data is presented as mean ± Standard Deviation (SD). Control group is represented by the bars not filled with dots and melatonin treated group is represented with bars are filled in. Distinct lowercase letters denote statistically significant differences between treatments determined using *t*-test (*n* = 3) for each sampling date. Distinct uppercase letters denote statistically significant differences between conditions determined using one-way analysis of variance (ANOVA) followed by Tukey’s multiple range test (*n* = 3) for each sampling date. A significance level of *p* < 0.05 was set for both statistical analyses.

The HPLC-MS/MS analysis allowed the identification of three anthocyanins in sweet cherry: cyanidin 3-*O*-rutinoside, cyanidin 3-*O*-rutinoside (isomer), and peonidin 3-*O*-rutinoside (Figure 2).

The quantification of individual anthocyanins revealed cyanidin 3-*O*-rutinoside as the predominant compound (70.20–91.42 mg/100 g), followed by its isomer (7.09–9.17 mg/100 g) and peonidin 3-*O*-rutinoside (3.67–5.12 mg/100 g). Regarding total anthocyanin content, fruits treated with melatonin at the preharvest stage showed significantly lower levels at harvest compared to control fruits (82.10 ± 0.24 vs. 88.55 ± 0.36 mg/100 g, respectively). After cold storage, control fruits exhibited a significant increase of 6% (93.71 ± 0.66 mg/100 g) compared to their initial content at harvest. In contrast, melatonin-treated fruits subjected to cold storage maintained its content when compared to these fruits at harvest (81.12 ± 0.37 mg/100 g), although its content was 13% lower compared to cold-stored control fruits. Regarding the postharvest application of melatonin followed by cold storage, control fruits did not show significant differences respect to control fruits at harvest (87.45 ± 3.20 mg/100 g). However, the combination of pre- and postharvest treatments with cold storage resulted in highest anthocyanins levels among all conditions studied (105.72 ± 0.54 mg/100 g), which led to a 29% increase compared to their values at harvest.

### 3.3. Impact of Melatonin Treatments on Hydroxycinnamic Acids Accumulation in Sweet Cherry Fruit

**Figure 3:**
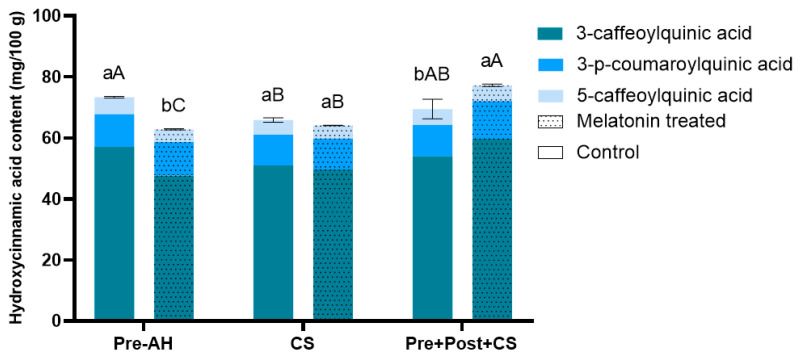
Hydroxycinnamic acid content (mg/100 g), specifically 3-caffeoylquinic acid, 3-*p*-coumaroylquinic acid, and 5-caffeoylquinic acid, in sweet cherries for the different treatments (control and melatonin) and conditions (preharvest: Pre-AH, cold storage: CS, and preharvest plus postharvest treatment followed by cold storage: Pre + Post + CS). Data is presented as mean ± Standard Deviation (SD). Control group is represented by the bars not filled with dots and melatonin treated group is represented with bars are filled in. Distinct lowercase letters denote statistically significant differences between treatments determined using *t*-test (*n* = 3) for each sampling date. Distinct uppercase letters denote statistically significant differences between conditions determined using one-way analysis of variance (ANOVA) followed by Tukey’s multiple range test (*n* = 3) for each sampling date. A significance level of *p* < 0.05 was set for both statistical analyses.

Regarding the levels of hydroxycinnamic acids, three major compounds were identified through the HPLC-MS/MS analysis: 3-caffeoylquinic acid, 3-*p*-coumaroylquinic acid, and 5-caffeoylquinic acid (Figure 3).

The quantification of individual hydroxycinnamic acids revealed 3-caffeoylquinic acid as the most representative acid (47.71–59.78 mg/100 g), followed by 3-*p*-coumaroylquinic acid (10.18–12.32 mg/100 g), and 5-caffeoylquinic acid (4.06–5.59 mg/100 g). In terms of total concentration, fruits treated with melatonin at the preharvest stage exhibited significant lower values of approximately 20% compared to control fruits (59.78 ± 0.22 vs. 75.17 ± 0.24 mg/100 g, respectively). After cold storage, control fruits showed a significant reduction of 10% in total hydroxycinnamic acid levels compared to their harvest values (67.66 ± 0.74 mg/100 g), while melatonin-treated fruits resulted in a slight but significant increase of 5% compared to those observed at harvest (62.60 ± 0.07 mg/100 g). Additionally, control fruits that received a postharvest treatment and were subsequently stored at low temperature exhibited similar levels than their harvest values, as no significant differences were found (74.63 ± 3.22 mg/100 g). Nevertheless, for melatonin-treated fruits, the postharvest melatonin application plus cold storage resulted in a significant increase of 30% compared melatonin-treated fruits at harvest (77.85 ± 0.40 mg/100 g), representing the highest values among all treatments tested.

### 3.4. Impact of Melatonin Treatments on Flavonol Accumulation in Sweet Cherry Fruit

**Figure 4:**
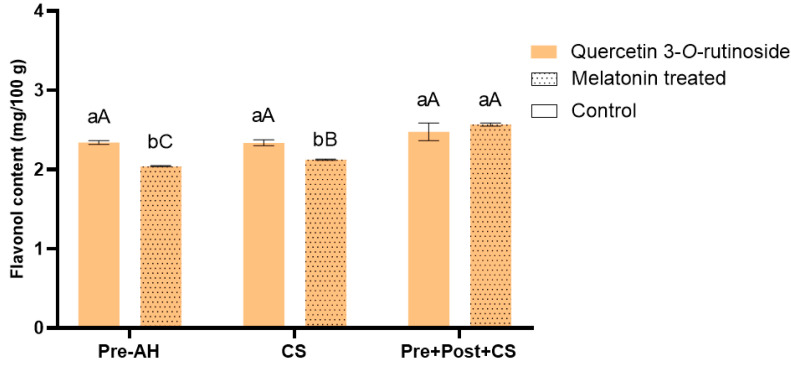
Flavonol content (mg/100 g), specifically quercetin 3-*O*-rutinoside, in sweet cherries for the different treatments (control and melatonin) and conditions (preharvest: Pre-AH, cold storage: CS, and preharvest plus postharvest treatment followed by cold storage: Pre + Post + CS). Data is presented as mean ± Standard Deviation (SD). Control group is represented by the bars not filled with dots and melatonin treated group is represented with bars are filled in. Distinct lowercase letters denote statistically significant differences between treatments determined using *t*-test (*n* = 3) for each sampling date. Distinct uppercase letters denote statistically significant differences between conditions determined using one-way analysis of variance (ANOVA) followed by Tukey’s multiple range test (*n* = 3) for each sampling date. A significance level of *p* < 0.05 was set for both statistical analyses.

The HPLC-MS/MS analysis enabled the identification of a single flavonols: quercetin 3-*O*-rutinoside (Figure 4).

The quantification revealed that preharvest-treated fruits had significant lower of approximately 19% compared to control fruits at harvest (1.95 ± 0.01 vs. 2.42 ± 0.02 mg/100 g, respectively). After cold storage, control fruits maintained similar levels to those observed at harvest since no significant differences were observed (2.39 ± 0.04 mg/100 g), while a significant increase of 7% was observed for melatonin-treated fruits (2.08 ± 0.01 mg/100 g). Postharvest treatment and subsequent cold storage maintained quercetin 3-*O*-rutinoside levels showed in control fruits at harvest (2.65 ± 0.11 mg/100 g), while the combination of pre- and postharvest melatonin treatments led to a significant increase of 32% (2.58 ± 0.02 mg/100 g) compared to their own harvest values, reaching values comparable to those of postharvest-treated control fruits and representing the highest among the all tested conditions.

### 3.5. Principal Component Analysis (PCA)

**Figure 5:**
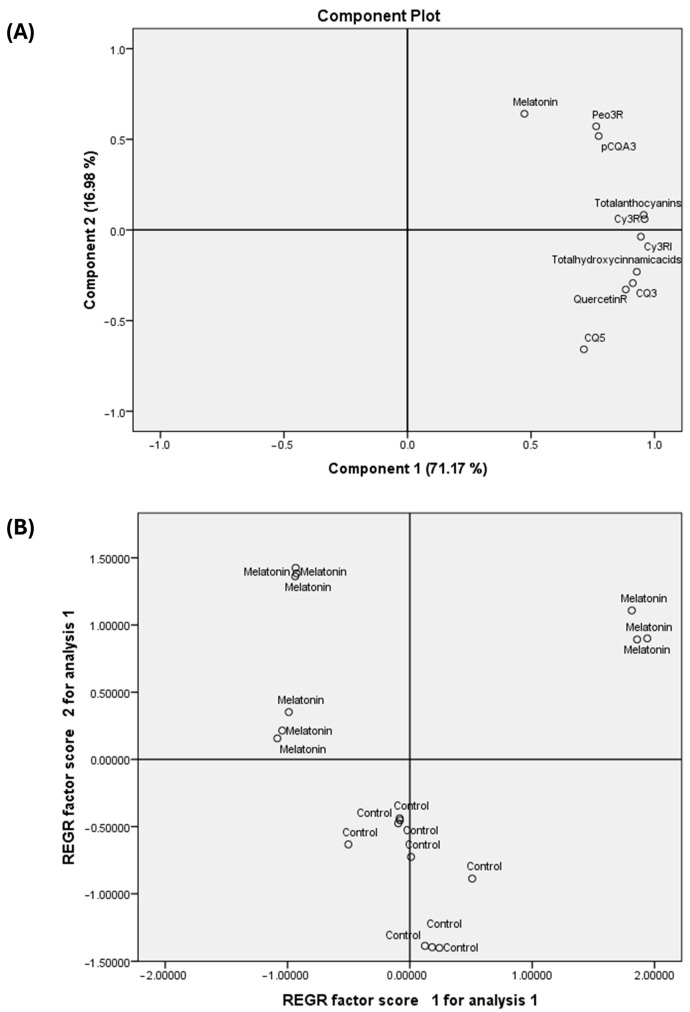
Principal component analysis (PCA) biplot showing the relationships among different parameters measured at different conditions (**A**) and treatments tested (**B**). The two principal components of the PCA explained 88.15% of the variation in the measured data. The variables measured were abbreviated as follows: cyanidin 3-*O*-rutinoside (Cy3R), cyanidin 3-*O*-rutinoside (isomer; Cy3RI), and peonidin 3-*O*-rutinoside (Peo3R), 3-caffeoylquinic acid (CQ3), 3-*p*-coumaroylquinic acid (pCQA3), 5-caffeoylquinic acid (CQ5) and the quercetin 3-*O*-rutinoside (QuercetinR). Treatments were abbreviated as follows: control and melatonin.

A Principal Component Analysis (PCA) was set to elucidate the possible relationship between the different parameters studied at different conditions and treatments applied (Figure 5).

The component plot (Figure 5A) from the PCA reveals the contribution of various variables to the first two principal components. Component **1** and Component **2** account for 71.17% and 16.98% of the total variance, respectively, explaining a cumulative 88.15% of the data’s variability. The plot shows that variables such as the content of total hydroxycinnamic acids, cyanidin 3-*O*-rutinoside (isomer), peonidin 3-*O*-rutinoside, quercetin 3-*O*-rutinoside, and 3-caffeoylquinic acid have high positive loadings on Component **1**, indicating that this component primarily represents a composite of these flavonoid and phenolic compounds. Melatonin exhibits a strong positive loading on Component **2** and a moderate positive loading on Component **1**, suggesting it is a significant contributor to both components and may have a distinct effect from the other compounds.

The factor score plot (Figure 5B) illustrates the clustering and separation of the experimental groups in the PCA space. The control group samples are tightly clustered around the origin of the plot, indicating low variance and a baseline profile. In stark contrast, the melatonin-treated samples are distributed across the right and top quadrants, displaying a clear and significant separation from the control group. The primary separation occurs along Component **1**, with the melatonin-treated samples consistently showing high positive scores. This distinct clustering provides strong evidence that the melatonin treatment induces a substantial change in the variables associated with Component **1**, particularly the phenolic compounds and flavonoids, while also causing a shift along Component **2**, where all melatonin samples possess positive scores.

## 4. Discussion

Melatonin has been extensively investigated in different sweet cherry cultivars due to its capacity to enhance fruit quality and confer health-promoting properties [27,35]. Beyond its well-known role in regulating circadian rhythm and sleep, melatonin exerts multiple beneficial effects in humans when consumed as a nutraceutical, including modulation of the immune response, strengthening of antioxidant defences, and improvement of cardiovascular function [36,37]. Recent evidence has also highlighted its neuroprotective role in delaying age-related cognitive decline, further expanding its health relevance when obtained through dietary sources [38,39]. Nevertheless, to our knowledge, the combinatorial effect of pre- and postharvest melatonin treatments combined with cold storage has not yet been investigated in a highly valued sweet cherry cultivar such as cv. ‘Sunburst’, particularly with respect to its impact on endogenous melatonin content.

Sweet cherries are naturally rich in melatonin, and its endogenous content is considered a relevant functional parameter, although this content is highly dependent on genetic background, environmental conditions, and agronomic practices. For instance, González-Gómez et al. [40] reported values ranging from 0.05 ng/100 g in ‘Ambrunés’ to 22.4 ng/100 g in ‘Burlat’ at harvest. The concentrations observed in ‘Sunburst’ in the present study fit within this range, although previous reports in the same cultivar have documented higher values between 10 and 20 ng/100 g [25,41]. These discrepancies may be attributed to differences in soil condition and agronomic practices, which can significantly influence fruit physiology [42,43]. The preharvest application of melatonin led to a clear increase on the endogenous melatonin content in sweet cherries at harvest. Previous research reported similar increases in ‘Samba’ and ‘Sandon Rose’ cultivars [44]. This response can be explained by direct absorption of the exogenous melatonin trough the physical infiltration [25]. Additionally, the activation of the melatonin biosynthetic pathway could be involved in this accumulation of endogenous melatonin, strengthening the fruit’s antioxidant network. For instance, several studies have reported that melatonin treatments could upregulate key biosynthetic genes, such as tryptophan decarboxylase, tryptamine 5-hydroxylase, serotonin *N*-acetyltransferase, and *N*-acetylserotonin methyltransferase, resulting in an enhanced biosynthesis of endogenous melatonin [20,44,45,46,47].

On the other hand, cold storage caused a marked reduction in the endogenous melatonin content in different fruits, regardless of melatonin treatment, consistent with previous observations in cherry tomatoes and lemons [21,46]. This decrease is likely related to melatonin’s role as a direct reactive oxygen species (ROS) scavenger and regulator of antioxidant enzyme activity under chilling stress, since low temperatures are a well-known abiotic stressor [48]. Melatonin is known to enhance the activity of enzymes, such as superoxide dismutase (SOD), catalase (CAT), and ascorbate peroxidase (APX), but this protective action can lead to a net depletion of its endogenous content during a prolonged stress [49]. Furthermore, during cold storage, sweet cherries continued its ripening process, which has been associated with a reduction in endogenous melatonin levels in this type of fruit [50,51]. This dual action, acting as both a stress mitigator and a molecule consumed during the defence response, provides a plausible explanation for the consistent decrease observed across storage conditions.

When melatonin was applied in postharvest and combined with cold storage (Control at Pre + Post + CS), endogenous melatonin levels were higher compared to control and preharvest-treated fruits under the same storage conditions (CS). This increase, which could be explained by the same mechanisms exposed previously, agreed with previously research in sweet cherries [18,25,52]. Moreover, the detrimental effect of chilling stress on melatonin content, as discussed above, was suppressed in sweet cherries that were treated after harvest. This suggests that immersion treatments promote a uniform distribution of melatonin throughout the fruit tissue, enabling direct absorption and the activation of the biosynthetic machinery [21,22]. The combined pre- and postharvest treatments followed by cold storage resulted in the highest endogenous melatonin content quantified among all conditions tested, highlighting a synergistic effect of this combination on the melatonin accumulation. The enhanced accumulation in this last condition could be explained due to cumulative exposure to the elicitor, which could extend the period of gene activation and maximizes the physical uptake. To our knowledge, this is the first study assessing the impact of combined pre- and postharvest melatonin treatments in conjunction with cold storage on the endogenous melatonin levels in sweet cherry fruit. To date, only two studies have employed a similar combinatory approach: Agulló et al. and Garrido-Auñón et al. [21,22] investigated ‘Fino’ lemons and ‘Sanguinelli’ blood oranges under analogous conditions and found that they had different effects on endogenous melatonin accumulation, depending on the fruit. These contrasting results emphasize the need to tailor melatonin application strategies to the physiological characteristics of each fruit species, highlighting the crop-specific nature of the response.

From a nutritional perspective, the enrichment of sweet cherries with melatonin may represent an additional benefit for consumers. Although it is a bioactive molecule with regulatory status as a dietary supplement, in this study its content was increased endogenously through pre- and postharvest treatments, likely by stimulating the fruit’s own biosynthesis pathways. Moreover, the concentrations observed in sweet cherries remain well below the intake limits of 2 and 10 mg per day established by European and American food and drug agencies, respectively [53]. Despite of those low amounts, the recurrent intake of melatonin-enriched fruits could contribute to sustained health benefits. Melatonin is a highly bioavailable molecule that is absorbed in the gastrointestinal tract, transported into the bloodstream, and capable of crossing the blood–brain barrier, thereby directly influencing systemic physiology [54]. Once incorporated, it acts as a potent antioxidant and free radical scavenger. It also upregulates endogenous antioxidant enzymes and modulates inflammatory signalling pathways, thereby contributing to mitochondrial protection and improved cellular homeostasis [54]. Moreover, regular consumption of melatonin-rich foods has been associated with improved sleep efficiency, regulation of circadian rhythms, and attenuation of age-related declines in pineal and tissue melatonin, which are particularly relevant in the context of insomnia and neurodegenerative disorders [38,55]. Beyond its well-documented antioxidant and sleep-promoting functions, dietary melatonin also could exert cardioprotective, anti-inflammatory, immunomodulatory, and neuroprotective effects, and may help reduce the risk of chronic diseases, such as diabetes, obesity, and cardiovascular dysfunction [55,56]. In this context, a recent randomized, placebo-controlled clinical trial demonstrated that melatonin supplementation (60 mg per day, administered for five days prior to surgery, in addition to standard care) ameliorated systemic inflammation and improved clinical outcomes in patients undergoing coronary artery bypass grafting, thereby highlighting its potential therapeutic role in cardiovascular interventions [57]. Therefore, the consumption of sweet cherries with an enhanced melatonin content could provide a natural and sustainable strategy to promote human health. Even if the concentrations in fruits are low compared to supplements, their recurrent dietary intake may exert cumulative effects over time, reinforcing antioxidant defences, protecting mitochondrial function, and supporting circadian health. This would offer a direct nutritional advantage to consumers and added value to cherry producers, as melatonin-enriched fruits could be marketed as foods with potentially enhanced bioactive composition.

In addition to melatonin, sweet cherries are characterized by a high content of phenolics compounds, which are key contributors to their health-promoting properties. Among these, the predominant family is anthocyanins, which are responsible not only for the fruit’s attractive red color, but also for a wide range of bioactive effects. These effects constitute a major commercial appeal for consumers [58,59,60]. Cyanidin-3-*O*-rutinoside, the most representative anthocyanin in sweet cherries, has been extensively studied for its biological activity. For instance, Zhong et al. [61] proposed its use as a potential strategy to mitigate dyslipidemia through the modulation of gut microbiota composition and lipid metabolism pathways. Furthermore, this compound may be linked to antidiabetic effects via stimulation of insulin secretion and to anti-obesity properties through the inhibition of lipid digestion and absorption [62,63]. Anthocyanins can also contribute to neuroprotection, anti-inflammatory responses, and vascular health, further consolidating their multifunctional role in human physiology and their high functional value [64,65,66]. Significant amounts of hydroxycinnamic acids were also reported, with 3-caffeoylquinic acid (chlorogenic acid) identified as the predominant compound in this study. Fu et al. [40] recently provided potential evidence supporting its pharmacological activity, particularly in promoting health through the regulation of gut microbiota [67]. In addition, chlorogenic acid has been associated with antioxidant, hepatoprotective, and cardiometabolic effects, which would make it a valuable contributor to the overall health-promoting profile of sweet cherries [68,69]. Lastly, quercetin-*O*-rutinoside (rutin), a flavonol recognized for its potential antioxidant, anti-inflammatory, anticancer, and cardioprotective effects, was detected [70]. However, flavonols occur at comparatively low levels in the present cultivar, so their overall contribution is expected to be smaller than that of anthocyanins and hydroxycinnamic acids.

At harvest, fruit that received the melatonin preharvest treatment showed lower levels of anthocyanins, hydroxycinnamic acids, and flavonols than controls. This pattern aligns with a ripening-delay effect, i.e., sweet cherries at a slightly less advanced developmental stage displays attenuated activation of the phenylpropanoid and flavonoid pathways [71]. Previous studies on sweet cherries suggest that anthocyanin accumulation during ripening is inhibited by regulatory factors, a finding that can be extrapolated to other phenolic compounds [51]. Furthermore, melatonin has a similar effect to auxins on plant growth regulation, delaying phenolic compound biosynthesis when it is applied in early stages [49,72,73]. Although classical maturity analyses were not conducted in the present study as it was not part of the main objective of it, it is well stablished that the progression of sweet cherry ripening is closely related to the accumulation of anthocyanins, which are responsible for the fruit’s characteristic reddish color. Therefore, the evaluation of anthocyanin accumulation in this study provides a reliable and biologically significant indicator for monitoring the dynamics of ripening.

Different phenolic compounds showed distinct trends during cold storage. In those cherries harvested from control trees, anthocyanin levels increased significantly, a response likely driven by both increased repining and cold-induced stress, which together could stimulate the synthesis of bioactive phenolics [16,71]. In contrast, melatonin-treated cherries maintained stable anthocyanin levels. This stability can be attributed to two protective mechanisms: (1) melatonin delays the fruit ripening process, slowing down the developmental activation of anthocyanin biosynthesis [51], and (2) treated fruits exhibit higher endogenous melatonin, which exerts strong antioxidant activity and reduces the need to synthesize anthocyanins as stress-responsive compounds [51,74]. This phenomenon could illustrate the capacity of melatonin to act as a central modulator of redox homeostasis, limiting unnecessary metabolic expenditure in secondary metabolism when antioxidant defences are already reinforced. As fruit ripens, the levels of hydroxycinnamic acids typically decline. During cold storage, the levels of these acids also decreased in the control cherries, which is consistent with their known reduction during ripening [75]. However, in melatonin-treated fruit, levels of hydroxycinnamic acids increased. This opposite result is theorized to be possible since the literature reports that melatonin upregulates genes that code for enzymes involved in the biosynthesis of hydroxycinnamic acids, such as phenylalanine ammonia-lyase, cinnamate-4-hydroxylase, hydroxycinnamoyl-CoA, and quinate hydroxycinnamoyl transferase. This effectively could enhance the levels of hydroxycinnamic acids under cold storage conditions [23]. Lastly, flavonol levels rose in both control and treated fruits during cold storage. This general increase may reflect cold stress–induced activation of the flavonoid biosynthetic pathway [76]. In treated fruit, melatonin could be involved in this response by promoting transcriptional flavonol enzymes and related regulatory genes [77].

When melatonin was applied at postharvest and followed by cold storage, the treatment did not significantly change anthocyanin, hydroxycinnamic acid, or flavonol concentrations compared with the control at harvest. This aligns with the previously discussed ripening-stabilizing effect, whereby modulation of ripening progression and attenuation of stress responses maintain phenolic levels close to their initial values rather than promoting further accumulation [51,74]. Moreover, this treatment resulted in lower anthocyanin levels than in control fruit after cold storage conditions, further supporting the effect of melatonin in delaying ripening and the associated synthesis of these pigments. Lower doses of melatonin may play an inhibitory role on sweet cherry ripening, obtaining lower anthocyanins levels after a cold storage since melatonin has a similar effect to auxins on the plant growth regulation [51,72]. Although another reports did not claim this reduction in anthocyanin levels after exogenous melatonin treatments, they also support a delay in the postharvest ripening and senescence of sweet cherries due to reduction on weight loss and decay incidence because of the increased antioxidant enzyme activity of those enzymes, such as CAT or APX, as well as the reduction in the H_2_O_2_ levels and malondialdehyde (MDA) content after the cold storage [18,25].

The combined application of preharvest and postharvest treatments followed by cold storage resulted in the highest concentrations of all phenolic compounds quantified. This outcome could be attributed to the prolonged exposure of sweet cherries to melatonin, which not only modulates fruit ripening but also plays a pivotal role in the activation of genes involved in the phenolic biosynthesis. In this sense, Michaidilis et al. [28] reported that the combinatorial pre- and postharvest treatment with melatonin at 0.5 mM could increase the transcript expression of 4-coumarate:CoA ligase 1, innamate-4-hydroxylase and dihydroflavonol 4-reductase of sweet cherry cv. ‘Ferrivia’. These enzymes are involved in the phenolics biosynthesis and can stimulate the phenylpropanoid pathway by the up-regulation of the transcript expression of the phenylalanine ammonialyase gene [28]. Thereby, an increase in the synthesis of flavonoids could occur [18,28,52]. Similar effects have been reported in other fruit species, such as lemons and blood oranges, where this combinatory approach enhanced the accumulation of secondary metabolites [21,22]. The synergy between extended elicitor exposure and cold storage could amplify these transcriptional responses, leading to a cumulative increase in the phenolic profile [26].

From a functional perspective, enhancing the levels of bioactive compounds, such as anthocyanins, hydroxycinnamic acids, flavonols, and melatonin in sweet cherries could provide dual benefits: (1) increasing their nutraceutical potential, and supporting antioxidant capacity, anti-inflammatory defences, metabolic regulation, and neuroprotection in consumers, and (2) raising the commercial value of cherries by positioning them as functional foods with potentially added health benefits. This dual role emphasizes the importance of pre- and postharvest melatonin strategies for maintaining fruit quality and shelf life, as well as responding to the growing demand for health-promoting foods.

These findings provide a practical, eco-friendly postharvest roadmap where preharvest spraying combined with postharvest immersion and standard cold storage (Pre + Post + CS) can produce cherries with enhanced endogenous melatonin and a strengthened phenolic profile, thereby increasing their potential health value and market appeal. This strategy would ensure not only improved fruit quality but also the enrichment of compounds with better nutraceutical properties, positioning sweet cherries as a functional food with added value for consumers. Importantly, the combined approach exploits both direct uptake of exogenous melatonin and transcriptional enhancement of secondary metabolism, achieving superior levels of melatonin, anthocyanins, hydroxycinnamic acids, and flavonols compared to single treatments. Furthermore, such optimized melatonin-based strategies could contribute to reducing postharvest losses, expanding export potential, and differentiating cherries in a competitive market through their positioning as premium functional foods. Although the 0.5 mM melatonin dose used in this study was selected based on previous literature reporting optimal effects on the biosynthesis of phenolic compounds, the response to melatonin is highly dependent on cultivar and dose. Thereby, it underscores the importance of fine-tuning treatments. A balance must be reached between delaying ripening, which can lead to reduced anthocyanin accumulation at harvest, and maximizing the synthesis and retention of bioactive compounds at postharvest. This compensation drives further research to investigate optimal protocols depending on the genetic background of each cultivar and commercial destination: immediate consumption or extended storage. The shelf life of early cherries is limited to not more than 10 days, so pre- and postharvest strategies to delay spoilage and maintain quality should be developed to minimize economical losses, especially for long exportations where fruit quality and functional properties can be affected [78].

## 5. Conclusions

This study evaluated for the first time the combinatorial use of pre and postharvest melatonin treatments followed by cold storage on a highly appreciated sweet cherry cultivar (‘Sunburst’). Preharvest melatonin application increased the endogenous melatonin levels but attenuated the accumulation of phenolics at harvest, reflecting a ripening delay effect. During cold storage, control fruits showed the expected rise in anthocyanins and decline in the hydroxycinnamic acids content, whereas melatonin-treated fruits maintained stable anthocyanins, accumulated hydroxycinnamic acids, and exhibited enhanced flavonol levels, highlighting the protective role of melatonin in modulating both ripening progression and stress responses. On the other hand, the postharvest application alone stabilized phenolic concentrations close to harvest levels, preventing further accumulation and reinforcing the ripening-stabilizing effect. Remarkably, the optimal treatment was found to be a combination of pre- and postharvest applications followed by cold storage. It produced the highest levels of both endogenous melatonin and phenolic compounds, indicating a synergistic effect derived from prolonged elicitor exposure. These findings provide the first integrated evidence that sequential pre- and postharvest melatonin treatments, combined with cold storage, represent a novel and eco-friendly approach to enhance sweet cherries health-promoting properties. This strategy delays ripening, maintains fruit quality, and could enrich the fruit’s bioactive composition. This opens new possibilities for improving both the health benefits for consumers and the commercial value of this fruit. Further studies should focus on defining optimal dose–response relationships and assess the influence of cultivar genetics on treatment efficacy. In parallel, genetic and transcriptomic studies are needed to validate the regulatory effects of melatonin on phenylpropanoid and flavonoid biosynthetic pathways, thereby strengthening the mechanistic basis of the observed outcomes. Finally, dedicated nutritional and clinical research is needed to investigate whether the enhanced accumulation of melatonin and phenolic compounds in sweet cherries effectively translates into measurable health benefits for humans.

## Data Availability

The original contributions presented in the study are included in the article, further inquiries can be directed to the corresponding author.
